# Oxygen and Glucose Levels in Cell Culture Media Determine Resveratrol’s Effects on Growth, Hydrogen Peroxide Production, and Mitochondrial Dynamics

**DOI:** 10.3390/antiox7110157

**Published:** 2018-11-05

**Authors:** Joao Fonseca, Fereshteh Moradi, Andrew J. F. Valente, Jeffrey A. Stuart

**Affiliations:** Department of Biological Sciences, Brock University, St. Catharines, ON L2S 3A1, Canada; jf14ms@brocku.ca (J.F.); fm15ta@brocku.ca (F.M.); valentaj@gmail.com (A.J.F.V.)

**Keywords:** Warburg, mitochondria, resveratrol, metabolism, glycolysis, reactive oxygen species, mitochondrial dynamics

## Abstract

Resveratrol is a plant-derived polyphenol that has been widely studied for its putative health promoting effects. Many of those studies have been conducted in cell culture, in supra-physiological levels of oxygen and glucose. Resveratrol interacts with reactive oxygen species (ROS) as an antioxidant or pro-oxidant. Resveratrol affects the expression and activities of ROS-producing enzymes and organelles. It is therefore important to consider how cell culture conditions might determine the effects of resveratrol on cultured cells. We determined the effects of resveratrol on cell growth, hydrogen peroxide production, and mitochondrial network characteristics in C2C12 mouse myoblasts and PC3 human prostate cancer cells under conditions of physiological (5%) and supra-physiological (18%) oxygen, and normo- (5 mM) and hyper-glycemia (25 mM). Interestingly, most effects of resveratrol on the parameters measured here were dependent upon prevailing oxygen and glucose levels during the experiment. Many of the effects of resveratrol on cell growth, hydrogen peroxide production, and mitochondrial network characteristics that were seen in 25 mM glucose and/or 18% oxygen were absent under the physiologically relevant conditions of 5 mM glucose with 5% oxygen. These findings emphasize the importance of using physiologically meaningful starting conditions for cell-culture experiments with resveratrol and indeed any manipulation affecting ROS metabolism and mitochondria.

## 1. Introduction

Reactive oxygen species (ROS), such as hydrogen peroxide (H_2_O_2_), are a normal product of cellular activity with important roles in cellular functions like mitosis, differentiation, and mitochondrial dynamics. On the other hand, abnormally high cellular ROS levels promote structural and functional changes in key marcromolecules including proteins and DNA. However, in certain pathological conditions, such as cancer and neurodegeneration, ROS homeostasis may be impaired [1]. Small molecules that can modulate cellular ROS metabolism therefore have therapeutic potential and have attracted significant interest.

Resveratrol (RES), a natural polyphenolic compound, can affect cellular ROS metabolism, either directly as an antioxidant or pro-oxidant [2,3], or indirectly by regulating expression of ROS-producing enzymes/organelles or antioxidant enzymes [4]. In the former instance, it appears that the concentration of RES used in cell culture experiments determines whether it behaves as a pro- or antioxidant [5]. In the latter case, RES treatment of cells in culture can affect ROS metabolism by increasing mitochondrial abundance [6,7] and regulating the expression and/or activity of NADPH oxidases (NOXs) [8] and nitric oxide synthases (NOSs) [9].

RES effects on animal cells are virtually always studied under standard cell culture conditions in which incubator O_2_ levels are not regulated, and headspace O_2_ is approximately 18%. This O_2_ level is substantially hyperoxic compared to in vivo [10], where the O_2_ levels experienced by most tissues cells are in the range of 1–6% [11]. All cells that we have tested previously produce more H_2_O_2_ at 18% O_2_ compared to 5% O_2_, and NOX isoforms 1 and/or 4 appear to be major contributors to this difference [12]. Given that RES interacts at multiple levels with cellular ROS metabolism it may be problematic to study RES effects under conditions of supra-physiological O_2_—there is a danger of measuring effects of RES that would not occur under physiologically relevant O_2_ levels. 

In addition to being hyperoxic, standard cell culture conditions are also typically hyperglycemic. Standard Dulbecco’s Modified Eagles Medium (DMEM) includes 25 mM glucose, which is approximately 5-times higher than normal plasma (glucose) in a healthy human. A robust effect of RES is its ability to promote a metabolic shift away from glucose fermentation and towards oxidative phosphorylation [13]. We have previously shown that many of RES’s effects on mitochondria observed in high glucose DMEM, including effects on mitochondrial dynamics, are absent in DMEM with galactose, which promotes oxidative phosphorylation [14]. In addition, since mitochondrial fusion is modulated by redox modifications of key proteins [15], maintaining appropriate O_2_ and glucose levels may be important.

Given the issues outlined above, it is important to consider the extent to which cell culture conditions might influence the outcome of experiments designed to determine effects of RES on a wide variety of cellular activities. To address this issue, here we have studied effects of low micromolar RES on two commonly studied cell lines (C2C12 mouse myoblasts and PC3 human prostate cancer) in which we have previously demonstrated RES effects ([14,16]). Here we use four conditions: physiological O_2_ and glucose levels; physiological O_2_ and high glucose; supraphysiological O_2_ and physiological glucose; and supraphysiological O_2_ and high glucose. We show that RES’s effects on cell growth, cell cycle, H_2_O_2_ production and mitochondria network morphology are all dependent on media oxygen and glucose levels. 

## 2. Experimental Procedures

Dulbecco’s Modified Eagle Medium (DMEM) high glucose (4500 mg/L; 25 mM) or low glucose (900 mg/L; 5 mM) containing L-glutamine, sodium pyruvate and sodium bicarbonate (Cat. #D6429), supplement-free Dulbecco’s Modified Eagle Medium powdered media (Cat. #D5030), fetal bovine serum (Cat. #F1051), non-essential amino acids, penicillin/streptomycin solution, 0.25% trypsin/EDTA solution, bovine serum albumin (BSA), horseradish peroxidase (2KU; Cat. #P6140) and H_2_O_2_ were obtained from Sigma-Aldrich (St. Louis, MO, USA). *trans*-Resveratrol (item 70675) and Amplex Red reagent (10-acetyl-3,7-dehydroxyphenoxazine; item 10010469) were purchased from Cayman Chemical (Ann Arbor, MI, USA). Dimethylsufoxide (DMSO), Tissue culture dishes (100 × 20 mm & 60 × 15 mm) were obtained from Sarstedt, Inc. (Newton, SC, USA). MitoTracker Red CMXRos and Lipofectamine 2000 transfection reagent was purchased from Life. C2C12 and PC3 cells were purchased from American Type Culture Collection (Manassas, VA, USA). Propidium Iodide (PI)/RNase Staining Buffer was purchased from BD bioscience (Cat#550825, SanDiego, CA, USA). All other reagents were obtained from Sigma-Aldrich (St. Louis, MO, USA). 

### 2.1. Cell Lines and Culture Conditions

C2C12 and PC3 cells were cultured according to the distributor’s protocol in high or low glucose DMEM supplemented with 10% fetal bovine serum (FBS), 2× MEM nonessential amino acid solution, and penicillin (50 I.U./mL)/streptomycin (50 μg/mL) solution. All cells were cultured within a humidified 5% CO_2_ atmosphere at 37 °C in one of two Forma 3110 water-jacketed incubators with O_2_ control (ThermoFisher, Waltham, MA, USA) set to either 18% O_2_ (supraphysiological) or 5% O_2_ (physiological) levels. In all cases, media was stored in cell culture dishes in the appropriate incubator for at least 24 h prior to its use to ensure equilibration with ambient conditions. For fluorescence microscopy experiments conducted at 5% O_2_, oxygen levels in the on-stage incubator were regulated with in-flow of humidified 5% O_2_/5% CO_2_/90% N_2_ gas mix. 

After thawing, cells were cultured at different oxygen (5% O_2_ or 18% O_2_) and glucose (25 mM and 5 mM) conditions for at least three days before starting the experiments. Cell density and population doubling time were determined by hemocytometer counting using Trypan Blue exclusion to identify live cells. Resveratrol and vehicle control (DMSO) were added directly to the culture media, and both media and treatments were exchanged every day. All media, solutions and buffers used during cell culture were per-warmed to 37 °C in an Isotemp 110 water bath (Fisher Scientific, Mississauga, ON, Canada) for at least an hour prior to use. 

### 2.2. Cell Cycle Analysis

Cell cycle distribution was analyzed as in [14]. Briefly, cells (~5 × 10^5^) were removed from plates by trypsinization, washed once with PBS, and then cell pellets fixed with ice-cold ethanol (75% *v*/*v*) and incubated overnight at −20 °C. The following day the suspension was transferred to room temperature, centrifuged (5 min at 240× *g*), washed twice with ice-cold PBS, and then incubated with 0.5 mL Propidium Iodide (PI)/RNase Staining Buffer (BD Pharmingen, San Jose, CA, USA) in darkness for 15 min. DNA content (PI signal) of the cell suspensions was immediately analyzed using a BD Accuri C6 flow cytometer (BD Biosciences, USA). The percentages of cells in G0-G1, S, and G2-M phases were determined using the CFlow Plus software (BD Biosciences, San Jose, CA, USA).

### 2.3. Hydrogen Peroxide Efflux Determination

Cellular H_2_O_2_ efflux was measured as in [12], using an Amplex Red reagent (10-acetyl-3,7-dihydroxyphenoxazine)-based assay, in which the fluorescent oxidation product resorufin serves as a proxy for H_2_O_2_ levels ([17]). Briefly, cells were seeded in either 25 mM or 5 mM glucose and placed in either 18% O_2_ or 5% O_2_ incubator overnight, before starting the treatments. Treatments were conducted for 48 h and refreshed every day. Immediately prior to experiments, cells were washed and then incubated in Krebs-Ringer buffer (KRB; 135 mM NaCl, 5 mM KCl, 1 mM MgSO_4_, 0.4 mM K_2_HPO_4_, 20 mM HEPES, 5.5 mM glucose supplemented with 10% fetal bovine serum) containing freshly added Amplex Red reagent (50 μM) and horseradish peroxidase (0.1 units/mL). 

A standard curve for H_2_O_2_ (0 μM to 3 μM) was included with each experiment. Cells were incubated for 2 h in 0.5 mL of KRB buffer, after which the buffer was collected and resorufin fluorescence measured using excitation and emission wavelengths of 535 nm and 595 nm, respectively (Cary Eclipse fluorescence spectrophotometer, Agilent Technologies, Santa Clara, CA USA). Simultaneously, cells were trypsinized and counted with a hemocytometer. H_2_O_2_ efflux rates (μmol·h^−1^) were standardized to cell number.

### 2.4. Stable C2C12 and PC3 Emerald Fluorescent Protein-Labelled Mitochondria

The plasmid mEmeral-Mito-7 was a gift from Michael Davidson (Florida State University). The plasmid contains a kanamycin-resistance gene for bacterial selection and geneticin (G418)-resistance gene for mammalian cell selection. Plasmid DNA was initially isolated and purified from bacterial cultures via a plasmid DNA Miniprep Kit (Norgen Biotek, Thorold, ON, Canada). Plasmid DNA purity (260 nm/280 nm absorbance ratio) and concentration were assessed by using a NanoPhotometer instrument (Montreal Biotech Inc., Ville Saint Laurent, PQ, Canada). 

To create stable cell lines, PC3 and C2C12 cells were plated in a 24-well plate with a desired cell density that allow cells to reach ~80% confluency after 24 h. Briefly, cells were transfected with Lipofectamine 2000 reagent and different combinations of plasmid DNA:Lipofectamine reagent were used. After 24 h, stable transfected cells were selected with G418 for 10 days (G418 concentration was determined in a prior screening experiment). After 10 days of selection, the concentration of G418 in culture media was dropped to a maintenance concentration. To confirm mitochondrial localization of the mEFP, colocalization of mEFP signal with the mitochondria-targeted fluorescent dye MitoTracker RedCMXRos was detected using confocal microscopy. 

### 2.5. Fluorescence Microscopy

Fluorescence micrographs of live cells were obtained using a Carl Zeiss Axio Observer. Z1 inverted light/epifluorescence microscope equipped with ApoTome.2 optical sectioning and a Hamamatsu ORCA-Flash 4.0 V2 digital camera. C2C12 and PC3 cells were cultured on Matek 35mm poly-d-lysine-coated glass bottom culture dishes for 48h under the glucose and oxygen conditions indicated. Media was refreshed every 24 h. Cells were switched to phenol red free media at least an hour prior to imaging. Cells were viewed with a Plan-Apochromat 63x/1.40 Oil DIC M27 microscope objective. The microscope stage and objectives were maintained at 37 °C, with temperature control achieved through TempModule S-controlled stage heater and objective heater (PeCon, Erbach, Germany). A humidified 5% CO_2_ environment with either 18% or 5% O_2_ was also maintained throughout the experiments. Green fluorescence was detected using a fluorescence channel possessing excitation and emission wavelength filter sets of 450–490 nm and 500–550 nm, respectively. Both the intensity of fluorescence illumination and camera exposure time were held constant throughout all experiments. Z-stacks consisted of 20 slices, each 0.25 µm apart. Maximum intensity projections were generated for each stack using the Fiji distribution of ImageJ. 

### 2.6. Quantitative Analysis of Mitochondrial Morphology

Mitochondrial morphology was assessed with the Mitochondrial Network Analysis tool (MiNA; [18]). To improve contrast between all mitochondrial structures and background, several pre-processing steps, such as contrast limited adaptive histogram equalization (CLAHE), median filtering and ‘unsharp mask’ were used. It was previously assessed that this combination of pre-processing steps provided the most accurate results. In the processed imaged, fluorescent mitochondrial signal was subjected to thresholding in order to eliminate background signal, which could generate an artifact. The binary image was then converted to a skeleton image in which mitochondrial signal was converted to lines of one pixel in width. From the skeletonized mitochondrial signal, skeleton structures were classified as either individuals (skeletons without branching) or networks (skeletons containing at least one branching point). Mitochondrial footprint, the total area in the image consumed by signal was also assessed. At least 30 cells per condition were selected randomly from at least three separate experiments.

### 2.7. Statistics

Statistical analyses were performed using JASP version 9 software and consisted of analyses of variance (ANOVA). Between-subject factors were oxygen (5%, 18%), treatment (DMSO, RES), and glucose level (Low, High). Alpha was set at *p* < 0.05, and post-hoc analyses consisted of independent-sample *t*-tests. All data are presented as means ± standard error of the mean (SEM). 

## 3. Results

**Figure 1:**
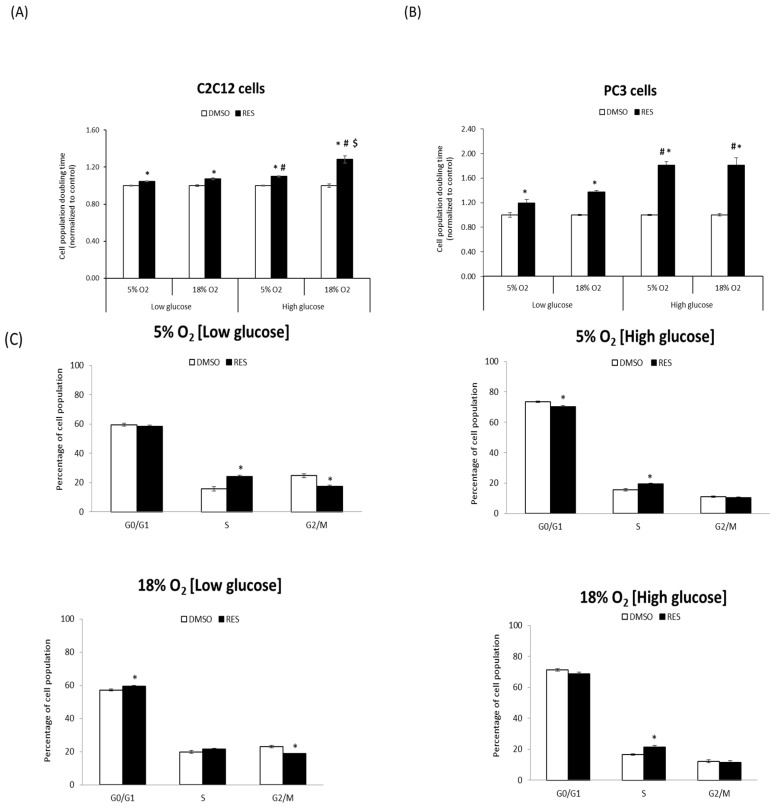
Media oxygen and glucose levels determine resveratrol’s effects on cell growth. Cell doubling time was measured following 48 h treatment with either Resveratrol (RES) (10 µM; filled bars) or vehicle control (DMSO; open bars). C2C12 (**A**) and PC3 (**B**) cells were cultured at either 5% O_2_ or 18% O_2_ in either low (5 mM) or high (25 mM) glucose Dulbecco’s Modified Eagles Medium (DMEM). (**C**) Distribution of C2C12 cells in different cell cycle stages was determined by flow cytometry. Data shown are means ± SEM of at least 3 independent experiments with *n* = 24 for cell doubling time (**A**,**B**) and *n* = 12 for cell cycle measurements (**C**). ‘*’ represents differences between RES-treated and DMSO-treated cells, *p* < 0.05. ‘#’ represents differences between low and high glucose levels, *p* < 0.05. ‘$’ represents differences between low and high oxygen levels.

One of the most robust effects of RES is the inhibition of cell proliferation. We investigated whether RES’s antiproliferative effects are influenced by O_2_ and glucose levels. For these experiments, C2C12 and PC3 cells were cultured at either 5% O_2_ or 18% O_2_ in either low (5 mM) or high (25 mM) glucose DMEM. C2C12 cell growth rates were strongly affected by media O_2_ and glucose levels, with lower O_2_ and lower glucose both favoring more rapid growth (Figure 1A). The effect of RES on C2C12 growth was dependent upon media conditions during the experiment: in cells growing in high glucose medium at 18% O_2_, 48 h treatment with 10 µM RES inhibited growth and increased cell population doubling time by almost 25% (Figure 1A). However, at 5% O_2_ and high glucose RES increased C2C12 population doubling time by only about 10%. Media glucose levels also modulated RES’s effects on proliferative cell growth. The ability of RES to slow growth was reduced in low glucose. In contrast with C2C12 cells, neither O_2_ nor glucose levels affected PC3 cell growth rates under control conditions (Figure 1B). However, RES more strongly inhibited growth in high glucose medium compared to low glucose (Figure 1B). To better understand the nature of RES’s O_2_- and glucose-dependent effects on C2C12 cell growth, we measured its effects on cell cycle distribution under the same four conditions. Under control conditions, low glucose media increased the proportion of cells in S and/or G2/M phase (Figure 1C), regardless of the O_2_ level. RES effects on cell cycle distribution were subtly affected by O_2_ or glucose levels; under all conditions RES treatment caused and increase, or a trend toward increase, in the proportion of cells in S-phase (Figure 1C). Taken together, these results indicate that O_2_ and glucose levels interact with RES’s effects on C2C12 cell growth.

**Figure 2:**
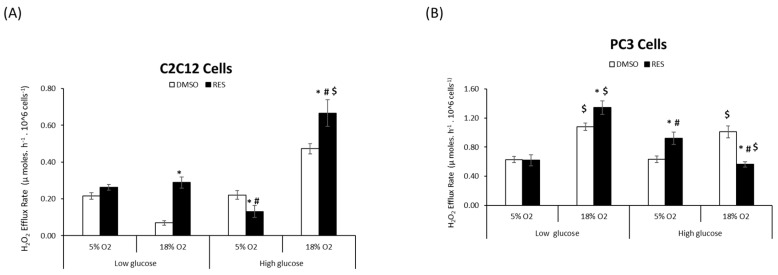
Media oxygen and glucose levels determine resveratrol’s effects on cellular hydrogen peroxide production. C2C12 (**A**) and PC3 (**B**) cells were cultured at either 5% O_2_ or 18% O_2_ in either low (5 mM) or high (25 mM) glucose DMEM. RES effects on H_2_O_2_ production were modulated by oxygen and glucose levels in both C2C12 (**A**) and PC3 (**B**) cells. In all experiments, cells were treated for 48 h with 10 µM RES (filled bars) or an equal volume of vehicle control (DMSO; open bars). Data shown are means ± SEM of at least 3 independent experiments (*n* = 12). ‘*’ represents differences between RES-treated and DMSO-treated cells, *p* < 0.05. ‘#’ represents differences between low and high glucose levels, *p* < 0.05. ‘$’ represents differences between low and high oxygen levels.

RES affects cellular ROS metabolism via its chemical antioxidant/pro-oxidant activities [4], its ability to stimulate mitochondrial respiration and biogenesis, [19], as well as the expression of ROS-producing enzymes such as NOX [8,20]. We investigated whether O_2_ and glucose interact with these RES effects by measuring cellular H_2_O_2_ production under all four O_2_ and glucose combinations. For C2C12 cells we found a main effect of each of the three variables glucose, oxygen, and treatment (RES) on H_2_O_2_ production (Figure 2A). The highest rates of H_2_O_2_ production under control conditions were seen in high glucose/high O_2_, while low glucose/high O_2_ resulted in the lowest rates of production_._ Interactions between oxygen*glucose and oxygen*treatment (*p* < 0.05) were also observed (Figure 2A). Overall, RES increased H_2_O_2_ production at 18% O_2_ irrespective of glucose level but had no effect on cells growing at 5% O_2_ (Figure 2A). Importantly, however, at physiological O_2_ and glucose levels, RES had no effect on H_2_O_2_ production in C2C12 cells. 

In PC3 cells, H_2_O_2_ production was greater in high O_2_ for both glucose levels. Again, however, while RES had complex effects on H_2_O_2_ production in PC3 cells growing in supraphysiological O_2_ and/or glucose, there was no effect of RES under more physiologically appropriate conditions of low glucose/low O_2_ (Figure 2B).

**Figure 3:**
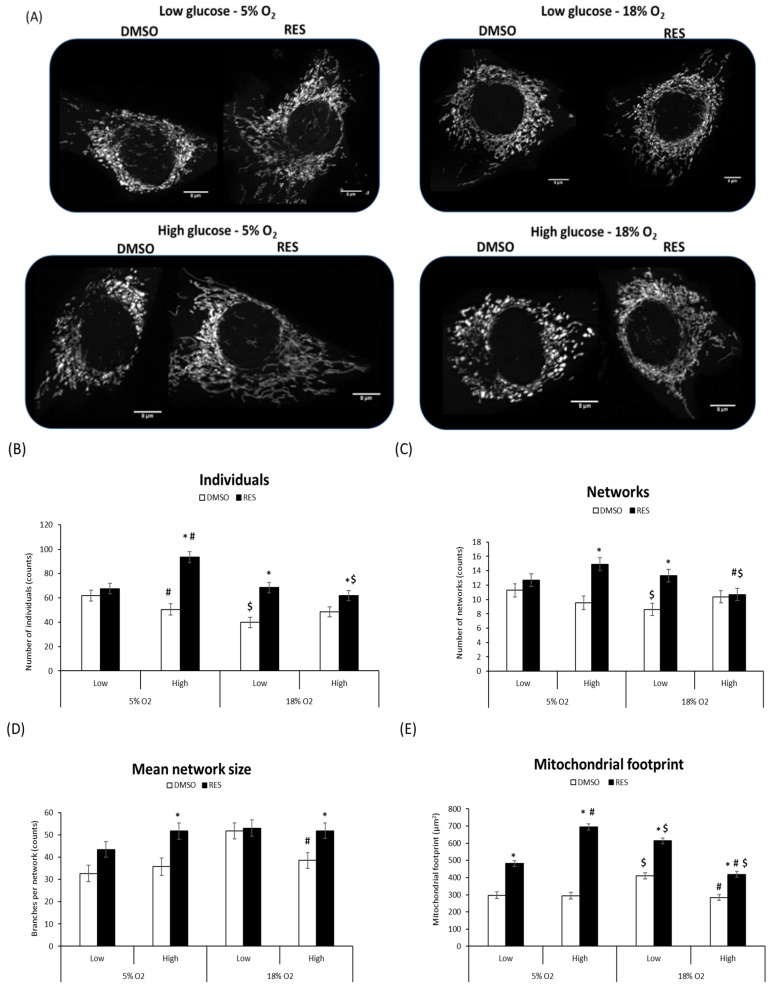
RES effects on mitochondrial network parameters are subtly affected by media glucose and oxygen levels in C2C12 cells. (**A**) Representative images of mitochondrial networks in C2C12 cells. There was a main effect of resveratrol on the number of individuals (**B**), networks (**C**), mean network size (branches per network) (**D**) and mitochondrial footprint (**E**). Overall, treatment with RES increased all these mitochondrial network parameters. An interaction between oxygen*glucose*treatment was also observed between individuals (**B**), number of networks (**C**) and mitochondrial footprint (**E**). A glucose and oxygen interaction was observed for mean network size (branches per network) (**D**). Total magnification in all experiments was 630×. Cells were treated for 48 h with 10 µM RES (filled bars) or an equal volume of vehicle control (DMSO; open bars). Data shown are means ± SEM of at least 3 independent experiments. For each condition at least 30 randomly selected individual cells were analyzed using MiNA. ‘*’ represents differences between RES-treated and DMSO-treated cells, *p* < 0.05. ‘#’ represents differences between low and high glucose levels, *p* < 0.05. ‘$’ represents differences between low and high oxygen levels.

**Figure 4:**
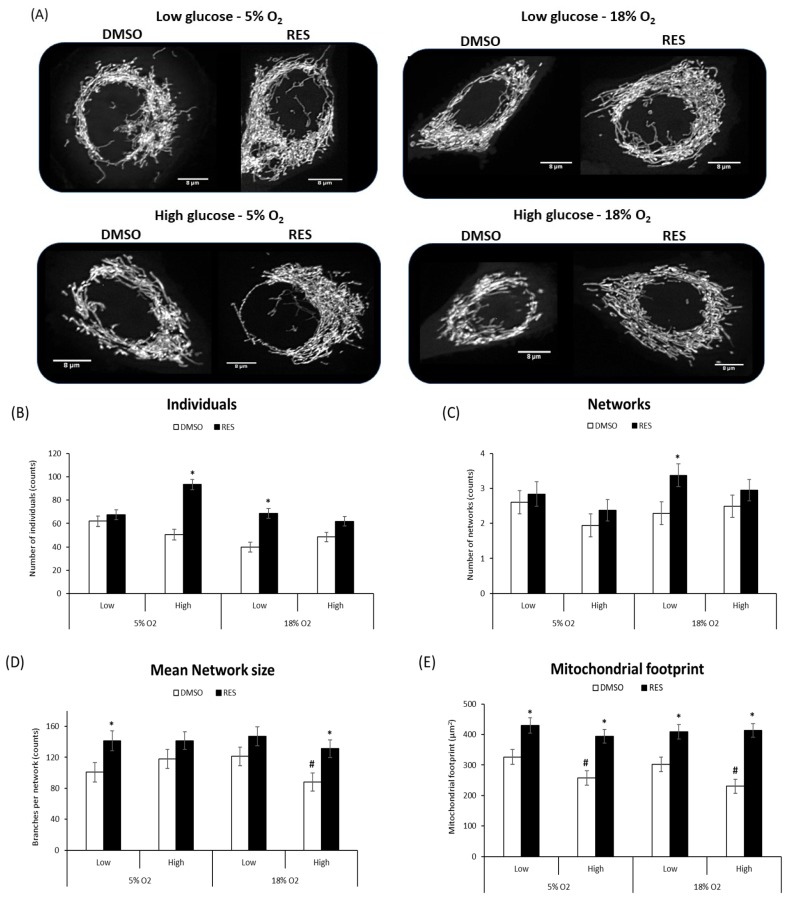
RES effects on mitochondrial network parameters are mostly unaffected by media glucose and oxygen levels in PC3 cells. Representative images of PC3 cells’ mitochondrial network parameters (**A**). There was a main effect of resveratrol on the number of individuals (**B**), networks (**C**), mean network size (branches per network) (**D**) and mitochondrial footprint (**E**). RES-treated cells showed an increase in all of these mitochondrial network parameters. An interaction between glucose and oxygen was also observed for mean network size (branches per network) (**C**). Main effect of glucose was observed in mitochondrial footprint (**D**). At low glucose levels mitochondrial footprint was increased. Total magnification in all experiments was 630×. Cells were treated for 48 h with 10 µM RES (filled bars) or an equal volume of vehicle control (DMSO; open bars). Data shown are means ± SEM of at least 3 independent experiments. For each condition at least 30 randomly selected individual cells were analyzed with MiNA. ‘*’ represents differences between RES-treated and DMSO-treated cells, *p* < 0.05. ‘#’ represents differences between low and high glucose levels, *p* < 0.05. ‘$’ represents differences between low and high oxygen levels.

Mitochondria and mitochondrial network characteristics are dependent on cytosolic redox state [15] and media glucose levels [21,22]. Therefore, interactions between RES, O_2_, and glucose would be expected to affect experimental outcomes. Indeed, we found that RES effects on mitochondrial network morphology in both C2C12 and PC3 cells were dependent on media glucose and oxygen levels (Figure 3 and Figure 4). Mitochondrial network characteristics were analyzed using the Mitochondrial Network Analysis tool (MiNA) that we recently developed and have described in detail elsewhere [18]. Briefly, MiNA can be used to identify and quantify features like ‘mitochondrial footprint’ (area of a 2D cell image occupied by mitochondria), the number of branched mitochondrial networks versus individual structures like rods or punctae, the number of individual branches per network, and the length of network branches or individual rod-shaped structures.

We found that O_2_ and glucose levels in culture affected mitochondrial network characteristics under control conditions. In low glucose and 18% O_2_, C2C12 cell mitochondria tended to be highly fused into relatively large networks and increased mitochondrial footprint. RES’s effects on mitochondrial network characteristics were influenced by these initial conditions. RES had no effect in C2C12 cells on the number of individual structures (rods and puncta), or the number of networks, when cells were growing in 5% O_2_ and low glucose, effects on these parameters were observed under other culture conditions (Figure 3B–D). In contrast, RES increased the mitochondrial footprint under all conditions (Figure 3E) and increased mean network size (number of branches) in most conditions. In PC3 cells, glucose and O_2_ levels had few effects in the absence of RES (Figure 4), though the mitochondrial footprint was consistently higher in low glucose media (Figure 4E). RES increased mitochondrial footprint under all conditions tested. In 5% O_2_ and low glucose, RES had no effect on the number of individual structures (rods and puncta) (Figure 4B), or the number of networks (Figure 4C), but increased mean network size (number of branches) (Figure 4D) in PC3 cells. Thus, under the most physiologically relevant conditions, RES promoted the production of larger and more highly branched networks in both C2C12 and PC3 cells.

## 4. Discussion

Most studies of RES’s molecular interactions within mammalian cells have been performed in cell culture inside humified incubators at 37 °C with CO_2_ regulated at 5% but no control over O_2_ levels, which are 18–19% under these conditions. Many cell culture media contain glucose at concentrations 2–5 times those found in normal human blood. Here we have demonstrated that these non-physiological conditions in cell culture can substantially affect the outcomes of these experiments.

The effects of RES on cell growth were strongly influenced by media glucose levels in both C2C12 cells and PC3 cells, with more subtle effects of RES observed in media with physiological glucose levels. Interestingly, the least effective inhibition of growth in both cell lines was observed in physiological O_2_ and glucose levels. This may be an important observation: although RES robustly inhibits growth of a wide variety of cancer cell lines in vitro [23,24,25], its effects on cancer growth in vivo are far less straightforward. This discrepancy has typically been attributed to low RES bioavailability in vivo [26,27], but our results suggest that an additional difference might be important: RES may simply be a less effective inhibitor of growth in physiologically normal O_2_ and glucose levels. This could relate to RES’s ability to switch cellular metabolism away from glucose fermentation and toward oxidative phosphorylation, and this switching can inhibit cancer cell growth [28,29]. Low glucose conditions already promote increased reliance on oxidative phosphorylation [22], so there may be limited further scope for RES to further affect this. In general, our results suggest that more attention should be paid to O_2_ and glucose levels during in vitro studies of cancer cell growth.

RES has multiple effects on cellular ROS metabolism, with chemical antioxidant and pro-oxidant activities, as well as effects on ROS production and ROS removal. We have found that high O_2_ levels in culture increase cellular H_2_O_2_ production [12]. This increased ROS production at high O_2_ might not create an ideal baseline from which to determine how RES affects ROS metabolism, since these conditions may not occur in vivo. Indeed, we found that, although both O_2_ and glucose interacted with RES in affecting cellular H_2_O_2_ production in C2C12 and PC3 cells, RES had essentially no effect when cells were growing in physiological O_2_ and glucose levels. Again, this emphasizes how these two parameters can determine experimental outcomes, which indicates the importance of maintaining both at physiologically appropriate values. If the antioxidant effect of RES is not observable in vivo, it may be because the effect is simply minimal or absent in the absence of high O_2_ or glucose.

We have previously shown that RES promotes fusion of the mitochondrial network [16]. However, mitochondrial dynamics are sensitive to redox state [15] and exposure to bolus H_2_O_2_ addition causes extensive fragmentation. It was therefore possible that the RES stimulation of mitochondrial fusion was simply reversing fragmentation caused by high O_2_. Our results did not support this, however; the effects of RES on mitochondrial network parameters seemed relatively independent of media glucose and O_2_. We found a main effect of RES on mitochondrial footprint, network size (number of branches), number of networks, and number of individual structures in both cell lines. The ability of RES to increase mitochondrial footprint and network size was observed even in low O_2_, low glucose media.

## 5. Conclusions

Overall, our results demonstrate the importance of O_2_ and glucose as determinants of RES’s effects on cell growth, ROS production, and mitochondrial network characteristics. Although we included only one cancer cell line here (PC3 cells), it will be important to determine whether the widespread observation that RES inhibits cancer cell growth in vitro can be replicated in more physiologically representative media. If not, this could offer a partial explanation for the failure of RES to achieve the same anti-cancer outcomes in vivo as have been seen in vitro. Similarly, we suggest that experiments to determine how RES affects cellular ROS metabolism should also be done in low O_2_ and low glucose media to ensure that outcomes properly represent what would occur in vivo.
